# Effects of Dual Modification with Succinylation and Annealing on Physicochemical, Thermal and Morphological Properties of Corn Starch

**DOI:** 10.3390/foods7090133

**Published:** 2018-08-28

**Authors:** Achmad Ridwan Ariyantoro, Nakako Katsuno, Takahisa Nishizu

**Affiliations:** 1 Department of Food Science and Technology, Universitas Sebelas Maret, Jalan Ir. Sutami No. 36 A, Kentingan, Surakarta 57126, Indonesia; ridwan030586@gmail.com; 2 The United Graduate School of Agricultural Science, Gifu University, 1-1 Yanagido, Gifu 501-1193, Japan; 3 Department of Applied Life Science, Gifu University, 1-1 Yanagido, Gifu 501-1193, Japan; nkatsuno@gifu-u.ac.jp

**Keywords:** annealing, corn starch, dual modification, succinylation

## Abstract

The objective of this study was to investigate the effects of annealing, succinylation, and a dual modification process (succinylation–annealing) on the physicochemical, thermal, and morphological properties of corn starch. Specifically, the properties of interest were the water-binding capacity (WBC), swelling power, paste clarity, solubility, pasting properties, stability ratio, and thermal and morphological characteristics. The dual modification process increased the physicochemical properties (WBC, swelling power, peak viscosity, and paste clarity) and increased the gelatinization temperature and gelatinization enthalpy (∆*H*), but had no effect on the morphological properties and X-ray diffraction patterns. A comparison of samples, made using each of the processes, showed that dual modification increased the stability ratio (more stable viscosity under thermal and shear stress), which was 0.69 for dual modified starch, compared with 0.64, 0.58 and 0.44 for native, succinylated, and annealed starches, respectively. The findings of the present study are of potential use in the food industry.

## 1. Introduction

Starch is an abundant organic substance that is useful to the food industry [1,2]. It has many applications, including as a stabilizer, gelling agent, and thickener [3]. Native starches have several weaknesses, such as low stability at high temperatures and under shear stress, thermal decomposition, and a high tendency for retrogradation [4,5]. Typically, modification processes are used to overcome these problems. Many modification methods have been used to improve starch properties, which can be divided into physical, chemical, and enzymatic processes [6].

Succinylation is a method that chemically modifies starch. It is an esterification reaction of a hydroxyl group in the starch molecule with succinic anhydride [7]. It results in higher viscosity, greater thickening power, and a lower retrogradation rate of starch [8]. Previous studies on modification by succinylation were conducted on yam starch [7], sorghum starch [8], corn starch [9], rice starch [10], and corn and amaranth starch [11]. The succinylation process confers many advantages, such as high solubility in cold water, high viscosity, better thickening power, increased paste clarity, retarded retrogradation, and freeze–thaw stability [10]. However, starch is still unstable during shearing at high temperatures [12]. Therefore, another modification process is required to overcome this weakness of the succinylation process. Annealing was chosen because of its ability to increase the thermal and shear stability of starch [13].

Unlike succinylation, annealing is a method of physically modifying starch. This process is widely used as, unlike chemical processes, it does not leave a residue [14]. Annealing modifies starch without damaging the granules [15,16,17]. It is generally performed above the glass transition temperature and below the gelatinization temperature, with high (>60%) or intermediate moisture content (40–50%) [18]. Annealing has been used as a modification process for various starch sources, and has been shown to decrease swelling power in numerous such sources [10,14,15,19,20,21,22,23]. Previous reports have also shown that annealing reduces breakdown viscosity (BD) [10,15,17,24]. A low BD and high stability ratio indicate high shear and temperature resistance of the starch [25,26]. Previous studies have shown that the stability ratio of native chestnut starch increased after annealing [27].

Recently, dual modification processes have been widely used to modify starch because they can improve its undesirable properties [5]. Various methods of dual modification have been widely reported, but there have been no reports on the dual succinylation–annealing process. Accordingly, no information is available with regard to the effects of this combined method on the physicochemical, morphological, and thermal properties of starch. Thus, the present study aimed to prepare starch using the succinylation–annealing method. The effects of annealing, succinylation, and dual modification (succinylation–annealing) on the physicochemical, morphological, and thermal properties of the starches were investigated. The effect of the stability ratio on heat and the shear stress resistance of these starches were also evaluated.

## 2. Materials and Methods

### 2.1. Materials

Corn starch (12.39% moisture content), succinic anhydride, and sodium carbonate were obtained from Nacalai Tesque, Inc. (Kyoto, Japan).

### 2.2. Succinylation Process

Succinylated starch was prepared by reacting native starch with succinic anhydride, with some modifications [8]. Starch (50 g) was added to 125 mL of distilled water with 1 g of sodium carbonate, and mixed with succinic anhydride (1 g). Succinylation was performed for 24 h at room temperature, with stirring using a magnetic stirrer. The mixture was filtered through filter paper (Whatman No. 1, GE Healthcare UK Ltd., Buckinghamshire, UK), and then washed with 20 mL of 95% ethanol. The precipitate was then washed with distilled water, and dried in an incubator (Eyela LTI-100ISD, Tokyo Rikakikai Co. Ltd., Tokyo, Japan) at 25 °C for 24 h. The dry starch was ground and sieved (100 mesh) to obtain succinylated starch using a cyclone sample mill (model 3010-018, UDY Corporation, Fort Collins, CO, USA).

### 2.3. Annealing Process

Annealing was performed according to the method described by Vamadevan et al. [28], with slight modifications. Starch (62.5 g) was added to 125 mL of distilled water and heated in a glass beaker using a water bath at 55 °C for 24 h. The starch suspension was then centrifuged (KN-70, Kubota, Osaka, Japan) at 1665× *g* to separate the water from the wet starch. The wet starch was dried in an incubator (Eyela LTI-100ISD, Tokyo Rikakikai Co. Ltd., Japan) at 25 °C for 24 h, ground, and sieved (100 mesh) to obtain annealed starch using a cyclone sample mill (model 3010-018, UDY Corporation, Fort Collins, CO, USA).

### 2.4. Dual Modification Process

Succinylated starch was obtained using the method described by Olayinka et al. [8], with some of the above-mentioned modifications, except for the drying process. Wet starch with a moisture content of about 40% was annealed and dried in an oven at 55 °C for 24 h. The dry starch was ground and sieved (100 mesh) using a cyclone sample mill (model 3010-018, UDY Corporation) to obtain dual modified starch.

### 2.5. Fourier-Transform Infrared (FTIR) Spectroscopy

FTIR spectra were recorded on an FTIR System (Spectrum 100 FTIR, Perkin Elmer, Waltham, MA, USA) using KBr pellets generated by way of the methodology described in Reference [29], with slight modifications. Starch (2 mg) was weighed, ground, and mixed uniformly with 200 mg of pure KBr powder. The starch was placed in an evacuable KBr die in a clear disk and then pressed using a hydraulic press. The sample was inserted into the FTIR system and scanned at a range of 400–4000 cm^−1^ to obtain a percentage of absorbance.

### 2.6. Degree of Substitution (DS)

The DS was determined using the alkali saponification method. A weight of 0.5 g of starch was added to a 100 mL conical flask with 25 mL of 75% ethanol. Subsequently, 20 mL of 0.5 M aqueous sodium hydroxide was added to the solution. The starch solution was stored at room temperature for 72 h, with occasional swirling of the flask. The excess alkali was back-titrated using 0.5 M hydrochloric acid [30]. The following equation was used to calculate the percentage of succinyl and DS:

(1) $$\%\mathrm{succinyl}=\frac{\left(\mathrm{blank}\;\mathrm{titre}-\mathrm{sample}\;\mathrm{titre}\right)\times 0.1\times \mathrm{molarity}\;\mathrm{of}\;\mathrm{acid}\times 100}{\mathrm{weight}\;\mathrm{of}\;\mathrm{sample}}$$

(2) $$\mathrm{Degree}\;\mathrm{of}\;\mathrm{substitution}\left(\mathrm{DS}\right)=\frac{162\times \%\mathrm{succinyl}}{10000-\left(99\times \text{\%}\mathrm{succinyl}\right)}$$

### 2.7. Water-Binding Capacity (WBC)

The WBC of the starch was determined using the method described by Abbey and Ibeh [31], with slight modifications. One gram of starch sample was weighed (W1) and added to 10 mL of distilled water at room temperature. The starch mixture was combined thoroughly using a vortex mixer for 30 s and centrifuged at 1665× *g* for 15 min. The supernatant and filtrate were separated, and the supernatant was weighed (W2). The WBC was expressed as g g^−1^ of starch.

(3) $$\mathrm{WBC}=\frac{W2-W1}{W1}$$

### 2.8. Swelling Power and Solubility

The swelling power was measured at 95 °C according to the method described by Waliszewki et al. [32], with slight modifications. The starch sample (5 mg) was added to a tube (W1). Distilled water (10 mL) was added to the tube, and the starch mixture was combined using a vortex mixer for 30 s. The starch suspension was heated in a water bath at 95 °C for 30 min. The mixture was cooled at room temperature and centrifuged at 1665× *g* for 15 min. The precipitate and supernatant were separated, and the precipitate was weighed (W2). The swelling power was measured using the following formula:

Swelling of starch = (W2 − W1)/weight of starch(4)

Solubility was measured using 5 mL of supernatant, which was dried in an oven at 110 °C for 24 h. The following formula was used to determine the solubility:

Solubility (%) = (weight of dry supernatant/weight of starch) × 100(5)

### 2.9. Paste Clarity

The paste clarity of native and modified starches was determined based on the method of Bhandari and Singhal [33]. Starch (5 mg) was added to a tube with 5 mL of distilled water. The starch suspension was heated at 95 °C for 30 min and shaken every 5 min. After cooling at room temperature, the percentage of transmittance of starch was measured using a spectrophotometer (Shimadzu model UV-3600, Shimadzu Corp., Kyoto, Japan) at 650 nm against a blank of water. Paste clarity was expressed as the percentage transmittance.

### 2.10. Pasting Properties

The pasting profiles were measured using a Rapid Visco Analyzer (RVA-Super 3, Newport Scientific, Warriewood, NSW, Australia) according to the procedure described by Wang et al. [14], with some modifications. Corn starch (3 g) was added into RVA canisters, and distilled water was added to make a total weight of 28 g. Peak viscosity (PV), BD, and trough viscosity (TV) were obtained from the RVA data. The relative breakdown was measured as the ratio of BD to PV [2], while the stability ratio was calculated as TV/PV [26]. All measurements were performed in triplicate.

### 2.11. Differential Scanning Calorimetry (DSC)

DSC measurements were conducted using a differential scanning calorimeter (Exstar SII-6200, Seiko Instruments Inc., Chiba City, Japan) based on the method of Wang et al. [34], with some modifications. Starch (3 mg) was weighed and added to aluminum pans. Distilled water (9 µL) was added to the aluminum pan using a syringe to obtain a starch-to-water ratio of 1:3 (*w*/*w*). The pans were sealed and allowed to stand for 30 min at room temperature prior to analysis. The samples were heated from 20 °C to 130 °C at a heating rate of 10 °C min^−1^. An empty aluminum pan was used as the reference. The gelatinization parameters were obtained using the Origin software (Version 2016, Microcal Inc., Northampton, MA, USA).

### 2.12. X-ray Diffraction Pattern

The X-ray diffraction patterns of native and modified starches were observed using an X-ray diffractometer (D8 Advance, Bruker AXS, Billerica, MA, USA), as described by Wang et al. [14]. The X-ray diffractometer was equipped with a copper X-ray tube operating at 40 kV and 40 mA. The starch samples were kept in a desiccator over a saturated solution of NaCl for 24 h prior to measurement. The diffraction intensity was measured from 10° to 30° as a function of 2θ, at a scanning speed of 1.67° min^−1^ and a step size of 0.04°.

### 2.13. Morphological Properties

The morphology of the starch granules was observed using the method of Thao and Noomhorn [35]. The shape of the starch granules was determined using a light microscope (BX53, Olympus, Tokyo, Japan) and cellSens Software, Version 2.1 (Olympus, Tokyo, Japan). Starch (0.1 g) was added to a tube containing 10 mL of distilled water and stirred thoroughly. A drop of starch suspension was placed onto a microscope slide and covered with a glass coverslip. The sample was observed at 1000× magnification.

### 2.14. Statistical Analyses

Data were analyzed statistically using the Origin software (Version 2016, OriginLab Corp., Northampton, MA, USA). Analysis of variance and Tukey tests with a significance threshold of *p* < 0.05 were used to compare the differences among mean values.

## 3. Results and Discussion

### 3.1. FTIR Spectroscopy and DS

**Figure 1:**
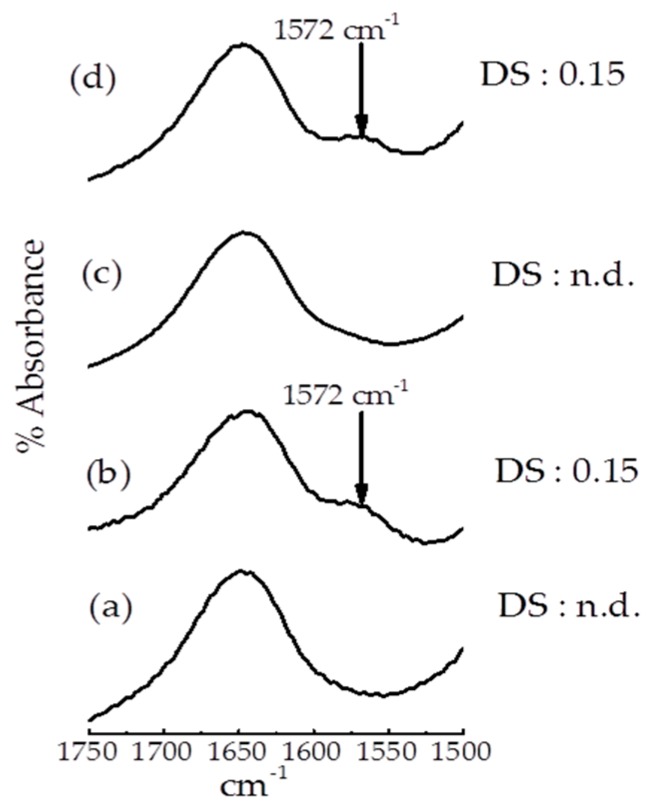
FTIR spectra for (**a**) native, (**b**) succinylated, (**c**) annealed, and (**d**) dual modified corn starch.

FTIR spectra and DS of native and modified of starches are shown in Figure 1. FTIR measurements can clarify whether succinylation was performed on the modified starch. The results of FTIR spectroscopy showed a small new peak at 1572 cm^−1^ with the succinylated and dual modified starches. The DS of the succinylated and dual modified starches were both 0.15, reflecting the fact that succinylation was also performed in the dual modified starch sample. The new peak at 1572 cm^−1^ suggested the existence of a carbonyl group (C–O) and that succinylation occurred in both the succinylated and dual modified samples. Consistent with previous studies, the asymmetric stretching vibration of carboxylate (RCOO^–^) of octenyl succinic anhydride starch was located at 1572 cm^−1^ [36,37].

### 3.2. Water Binding Capacity (WBC)

The WBC of native, succinylated, annealed, and dual modified corn starches are summarized in Table 1. The hydrophilic tendency of starch can be measured by its WBC [38]. The WBC of succinylated and dual modified starches were 0.84 and 0.83 g g^−1^, respectively, both of which were higher than that of the native starch (0.69 g g^−1^). Succinylation increased the water binding capacity of starch, compared to the native counterpart. Succinylation also resulted in a more pronounced hydrophilic tendency and expansion of some amorphous regions [39]. Moreover, Arueya et al. [39] stated that succinylation introduced bulky functional groups, and their electrostatic repulsion led to percolation and absorption of water within the starch matrices. There was no significant difference in the WBC of dual modified starch compared with succinylated starch.

Consistent with previous results in white sorghum starch [38], the WBC was decreased in the annealed starch compared with that of the native starch. The lower WBC in annealed starch than in native starch is due to a decrease in the number of available water-binding sites after annealing, caused by amylose–amylose and amylose–amylopectin interactions in the annealing process [38].

### 3.3. Swelling Power and Solubility

The swelling power and solubility values with different treatments are shown in Table 1. The swelling power increased slightly after the dual modification process, from 16.4 (native starch) to 17.7 g g^−1^. There was no significant difference between the swelling power of succinylated starch, native starch, and dual modified starch. However, annealing decreased the swelling power of the native starch. This finding is in agreement with previous reports on corn starch [14], barley starch [19], wheat starch [20], rice starch [15,21], oat starch [13], and sorghum starch [17,22,38].

The annealing treatment of corn starch resulted in a lower swelling power value than that of the native starch. Waduge et al. [19] reported that the swelling power decreased in the post-annealing process of barley starch. This decrease in swelling power was due to the interaction of crystallite perfection and the interplay of amylose–amylose during the annealing process.

Several factors influence the swelling power, such as the structure of amylopectin [40], complexes of V-amylose lipid [41], amylose content [42], enhancement of crystalline perfection [17,19], increment in molecular organization [23], and the level of interaction between amylose–amylose and/or amylose–amylopectin chains. There was no significant difference in the solubility of native, succinylated, annealed, and dual modified starches.

### 3.4. Paste Clarity

The paste clarity values of succinylated, annealed, and dual modified starches are presented in Table 1. The clarity of the starch pastes was increased following succinylation (72.9%), annealing (67.4%), and dual modification (68.6%) compared with the native starch (65.4%). Succinylation increased the paste clarity of corn starch compared with that of the native starch paste. A previous report by Bhandari and Singhal [11] showed the same result: Increased paste clarity of corn and amaranth starches after succinylation. The succinyl group is substituted for the hydroxyl groups on the starch molecules, inhibiting the formation of ordered structures after gelatinization and preventing retrogradation, resulting in a more transparent paste [43].

The paste clarity value increased slightly after annealing compared with the native starch. This result is consistent with results reported by Ali and Hasnain [38], in which annealing increased the paste clarity of white sorghum starch compared with that of native sorghum starch. Increased paste clarity of annealed sorghum starch was correlated with decreased solubility, and greater amylose leaching and amylopectin interaction led to a higher turbidity of the starch paste [44]. In the present study, there was no significant difference in paste clarity between dual modified starch and annealed starch.

### 3.5. Pasting Properties

The pasting properties of the four starches are summarized in Table 2. The succinylated and dual modified starches had higher PV values than the native starch, consistent with previous findings [8,10,45]. In a study on potato starch, Hui et al. [45] reported that the PV increased following octenyl succinic anhydride modification. The introduction of bulky hydrophilic succinate groups leads to increased starch chain expansion and PV [8,10]. In the present study, there was no significant difference between the PV values of annealed starch and the native starch.

The BD values of native, succinylated, and annealed starches were 970, 1697, and 1481 mPas, respectively. Succinylated starch had a higher BD than the native starch. Similar trends were observed in rice [10], yam [8], cocoyam [46], and corn starches [47]. Succinylation of starch leads to partial degradation as the integrity of the starch granules cannot be maintained. The decrement of granule integrity led to an increase in BD when heat and shear stress were applied [46].

Annealing enhanced BD compared with the native starch. This was consistent with a study by Adebowale et al. [22], which demonstrated that BD increased after annealing in sorghum starch. This indicates less stability at high temperatures and under shear stress. Hormdok and Noomhorm [21] also demonstrated that annealing increased BD in rice starch. The relative BD is defined as the ratio of BD to PV [2]. The relative BD of succinylated and annealed starch increased significantly, from 0.36 (native starch) to 0.42 and 0.56, respectively. However, the BD of dual modified starch was significantly lower than that of the other three samples. Moreover, the dual process significantly decreased the relative breakdown from 0.42 to 0.31 compared with succinylation.

The stability ratio indicates the resilience of starch to heat and shear stress [26], and is calculated as TV/PV. The stability ratio of succinylated and annealed starches was lower than that of the native starch. The stability ratio of dual modified starch increased from 0.58 (succinylated) to 0.69, suggesting that the process increased the stability ratio compared with succinylation. The interaction of succinylation and annealing resulted in good stability ratios. Succinylation led to an increase in PV, while annealing led to a decrease in BD. Succinylation occurred in the amorphous regions as they were more accessible to chemical reactions than the crystalline regions [8,48]. The introduction of the succinyl group increased the hydrophilic properties, leading to an increase in PV [8,10,39,43]. This hydrophilic tendency led to an increase in the WBC of the starch. Succinylated starch has a higher WBC value than annealed starch; therefore, succinylated starch can absorb more water than annealed starch.

Annealing is correlated with the physical reorganization of starch granules in the presence of water [49]. The presence of more water leads to an increased mobility of the amorphous regions to a crystalline state [50], resulting in the starch being more resistant to heat and shear stress, thus reducing the BD.

### 3.6. Thermal Properties

The characteristics of an endothermic transition were shown by each of the starches in DSC, which appear between 60 °C and 80 °C. Table 3 shows the DSC gelatinization parameters of these four starches. DSC results suggested that the gelatinization temperature increased following annealing. These results are similar to those of previous reports on normal corn starch [14], barley starch [19], wheat starch [24], and waxy barley starch [51]. The gelatinization temperature increased as a result of the enhanced perfection of crystallites [18,19,49,52,53].

There was no significant difference in enthalpy between native and annealed starch. No effect of annealing on enthalpy gelatinization (∆*H*) has previously been reported in corn starch [14,42,54], barley starch [19], rice starch [21], or wheat starch [24]. The lack of change in enthalpy gelatinization (∆*H*) in barley starch after annealing indicated that annealing did not form a new double helix [19], and there was no significant difference in gelatinization temperature and enthalpy gelatinization in corn starch after succinylation, compared with the native starch. However, dual modification of corn starch increased the temperature of the onset of the endothermic transition (*T*c) and ∆*H* compared with the native starch. There were no significant differences between annealing and succinylation with regard to gelatinization temperature and enthalpy.

### 3.7. X-ray Diffraction Pattern

**Figure 2:**
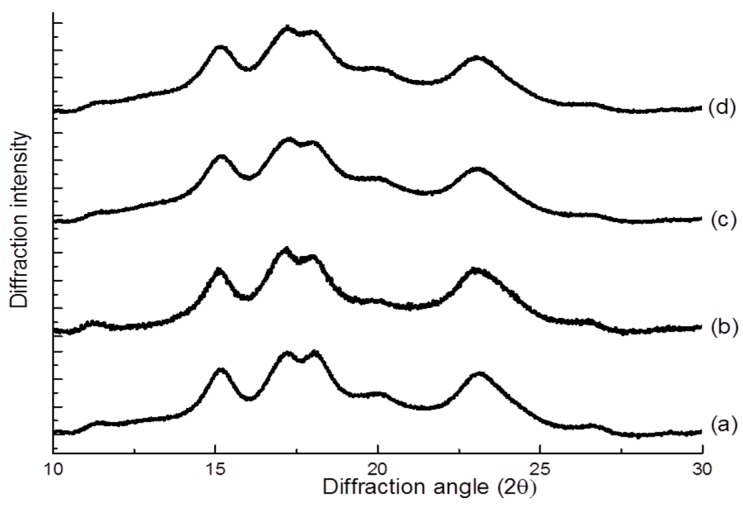
X-ray diffraction pattern for (**a**) native, (**b**) succinylated, (**c**) annealed, and (**d**) dual modified corn starch.

The X-ray diffraction patterns of the native and modified starches are presented in Figure 2. The native and modified starches showed characteristic A-type diffraction patterns, with two peaks at 15.1 and 23.2, and a double peak at 17.1 and 18.0 (2θ). These observations indicated that the annealing process did not alter the diffraction pattern of corn starch, consistent with previous findings [14,15,16,17,19,34,55,56]. Although annealing did not change the type of crystallinity pattern, it could increase the relative crystallinity [56]. Moreover, succinylation and dual modification did not change the diffraction patterns of starch.

### 3.8. Morphological Properties

**Figure 3:**
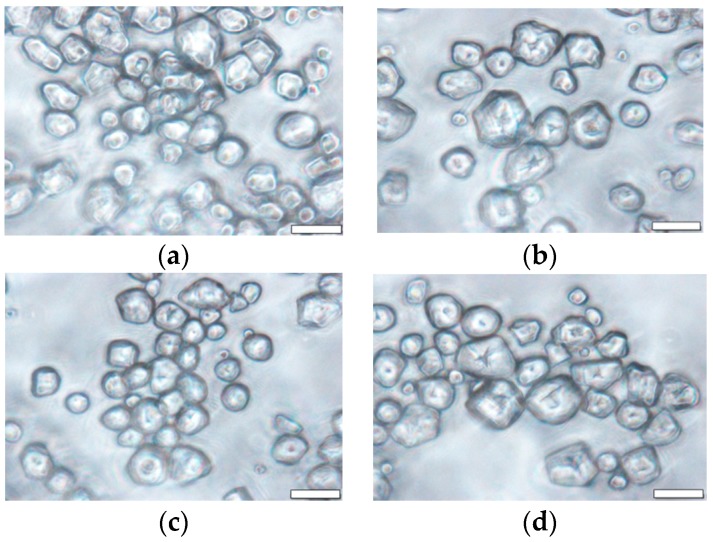
Micrographs of (**a**) native, (**b**) annealed, (**c**) succinylated, and (**d**) dual modified corn starch. Scale bar represents 20 µm.

Corn starch granules were observed under a light microscope, as shown in Figure 3. In agreement with previous reports from Wang et al. and Rocha et al. [14,16], corn starch granules showed polyhedral and rounded shapes. Succinylation did not change the granule shape compared with the native starch. These observations are similar to those reported by Arueya et al., Emeje et al., and Ayucitra [39,57,58]. Furthermore, the shape, appearance, and granule structure were not destroyed after succinylation. Likewise, the granule shape was unchanged after annealing compared with the native starch, consistent with previous findings [14,16,19,24,38,55,59].

Wang et al. [14] suggested that the granule morphology of corn starch did not significantly change after annealing. The granules showed a variety of shapes, with small granules being spherical and large granules being polyhedral. Rocha et al. [16] also reported that there were no differences in granule shapes after annealing. The granule shape of corn starch remained unchanged after dual modification. Dual modified starch also showed polyhedral and rounded shapes. This reveals that there is no difference between the shape of dual modified starch and native starch, and suggests that dual modification does not affect the shape and appearance of starch granules.

## 4. Conclusions

The preparation of dual modified corn starch was investigated. Our results show that dual modification increased the water binding capacity, swelling power, peak viscosity, paste clarity, gelatinization temperature, and enthalpy gelatinization, while it had no effect on solubility, granule shape, and X-ray diffraction pattern compared with the native starch. Dual modification was also shown to be more effective than succinylation at increasing the stability ratio. These results suggest that the undesirable properties of succinylated corn starch can be overcome by dual modification. The findings of this study are of potential use to the food industry in products, such as sauces that require starches that are resistant to heat and shear stress during processing. Further investigation into the use of this dual modification process is required for other types of starch.

## Figures and Tables

**Table 1:** Effects of annealing, succinylation, and dual modification on the physicochemical properties of starch.

Sample	WBC (g g^−1^)	Paste Clarity (%)	Swelling Power (g g^−1^)	Solubility (%)
Native	0.69 ± 0.03 ^b^	65.4 ± 0.5 ^c^	16.4 ± 0.2 ^b^	11.2 ± 0.2 ^a,b^
Succinylated	0.84 ± 0.01 ^a^	72.9 ± 1.3 ^a^	17.3 ± 0.2 ^a,b^	12.7 ± 1.2 ^a^
Annealed	0.66 ± 0.02 ^c^	67.4 ± 0.7 ^a,b^	14.2 ± 0.4 ^c^	9.7 ± 0.6 ^b^
Dual Modified	0.83 ± 0.01 ^a^	68.6 ± 0.5 ^b^	17.7 ± 0.4 ^a^	11.1 ± 0.8 ^a,b^

WBC: Water-binding capacity. Values represent the mean of triplicate measurements ± SD (Standard Deviation). Means within columns with different letters are significantly different (*p* < 0.05).

**Table 2:** Effects of annealing, succinylation, and dual modification on the pasting properties of starch.

Sample	Peak Viscosity (mPas)	Trough Viscosity (mPas)	Breakdown Viscosity (mPas)	Relative Breakdown	Stability Ratio
Native	2707 ± 43 ^c^	1737 ± 37 ^c^	970 ± 33 ^b^	0.36 ± 0.01 ^c^	0.64 ± 0.01 ^b^
Succinylated	4111 ± 258 ^a^	2414 ± 42 ^a^	1697 ± 165 ^a^	0.42 ± 0.02 ^b^	0.58 ± 0.02 ^c^
Annealed	2650 ± 49 ^c^	1168 ± 104 ^d^	1481 ± 31 ^a^	0.56 ± 0.01 ^a^	0.44 ± 0.01 ^d^
Dual modified	3171 ± 14 ^b^	2188 ± 20 ^b^	982 ± 13 ^b^	0.31 ± 0.004 ^d^	0.69 ± 0.004 ^a^

Values represent the mean of triplicate measurements ± SD. Means within columns with different letters are significantly different (*p* < 0.05).

**Table 3:** Effects of annealing, succinylation, and dual modification on the thermal properties of starch.

Sample	*T*o (°C)	*T*p (°C)	*T*c (°C)	∆*H* (mJ g^−1^)
Native	66.8 ± 0.2 ^a^	73.5 ± 0.5 ^a^	81.7 ± 0.3 ^b^	7.9 ± 0.3 ^b^
Succinylated	67.2 ± 1.1 ^a^	74.0 ± 0.4 ^a^	82.0 ± 0.05 ^a^^,^^b^	9.2 ± 0.9 ^a,b^
Annealed	70.9 ± 0.1 ^b^	75.4 ± 0.1 ^b^	82.4 ± 0.1 ^a,b^	8.2 ± 0.5 ^a,b^
Dual modified	67.6 ± 0.6 ^a^	74.1 ± 0.4 ^a^	82.6 ± 0.5 ^a^	9.7 ± 0.5 ^a^

*T*o, *T*p, and *T*c are the temperatures of the onset, peak, and conclusion of the endothermic transition, respectively. ∆*H* is the enthalpy gelatinization. Values represent the mean of triplicate measurements ± SD. Means within columns with different letters are significantly different (*p* < 0.05).
